# MicroRNA-587 antagonizes 5-FU-induced apoptosis and confers drug resistance by regulating PPP2R1B expression in colorectal cancer

**DOI:** 10.1038/cddis.2015.200

**Published:** 2015-08-06

**Authors:** Y Zhang, G Talmon, J Wang

**Affiliations:** 1 Eppley Institute for Research in Cancer and Allied Diseases, 985950 Nebraska Medical Center, Omaha, NE 68198, USA; 2 Department of Genetics, Cell Biology and Anatomy, 985950 Nebraska Medical Center, Omaha, NE 68198, USA; 3 Department of Pathology and Microbiology, University of Nebraska Medical Center, 985950 Nebraska Medical Center, Omaha, NE 68198, USA; 4 Department of Biochemistry and Molecular Biology, Fred & Pamela Buffett Cancer Center, 985950 Nebraska Medical Center, Omaha, NE 68198, USA

## Abstract

Drug resistance is one of the major hurdles for cancer treatment. However, the underlying mechanisms are still largely unknown and therapeutic options remain limited. In this study, we show that microRNA (miR)-587 confers resistance to 5-fluorouracil (5-FU)-induced apoptosis *in vitro* and reduces the potency of 5-FU in the inhibition of tumor growth in a mouse xenograft model *in vivo*. Further studies indicate that miR-587 modulates drug resistance through downregulation of expression of PPP2R1B, a regulatory subunit of the PP2A complex, which negatively regulates AKT activation. Knockdown of PPP2R1B expression increases AKT phosphorylation, which leads to elevated XIAP expression and enhanced 5-FU resistance; whereas rescue of PPP2R1B expression in miR-587-expressing cells decreases AKT phosphorylation/XIAP expression, re-sensitizing colon cancer cells to 5-FU-induced apoptosis. Moreover, a specific and potent AKT inhibitor, MK2206, reverses miR-587-conferred 5-FU resistance. Importantly, studies of colorectal cancer specimens indicate that the expression of miR-587 and PPP2R1B positively and inversely correlates with chemoresistance, respectively, in colorectal cancer. These findings indicate that the miR-587/PPP2R1B/pAKT/XIAP signaling axis has an important role in mediating response to chemotherapy in colorectal cancer. A major implication of our study is that inhibition of miR-587 or restoration of PPP2R1B expression may have significant therapeutic potential to overcome drug resistance in colorectal cancer patients and that the combined use of an AKT inhibitor with 5-FU may increase efficacy in colorectal cancer treatment.

Colorectal cancer is the third most common cancer and the second leading cause of cancer-related mortality in the US. 5-Fluorouracil (5-FU) is one of the chemotherapeutic drugs most widely used alone or combined with other drugs in colorectal cancer treatment.^1^ 5-FU primarily interrupts synthesis of the pyrimidine thymidine, a nucleoside required for DNA replication, by blocking the activity of thymidylate synthase.^2^ Consequently, 5-FU induces cell cycle arrest and/or apoptosis in cancer cells. Although adjuvant 5-FU treatment has yielded a good success rate, the failure of treatment in over 90% of patients with metastatic cancer is due to drug resistance.^3^ Many mechanisms have been suggested to be responsible for drug resistance, including blocking apoptosis.^2, 4, 5, 6^ Although resistance to chemotherapy is one of the biggest obstacles for effective cancer therapy, no significant advance has been made to identify targets overcoming drug resistance.^7^

MicroRNAs (miRNAs) are a class of small (about 22 bps) non-coding regulatory RNA molecules, which regulate gene expression primarily by binding to the 3′-UTRs of their target mRNAs to initiate sequence-specific mRNA cleavage or to inhibit translation.^8^ It is estimated that more than one-third of human genes and the majority of genetic pathways are regulated by miRNAs.^9^ MiRNAs have been virtually linked to all known biological processes as well as various pathological diseases including cancer.^10^ Alternations in miRNA expression have been associated with many human cancers.^11, 12^ The pleiotropic nature of gene regulation by miRNAs implies that some miRNAs may function as crucial mediators of drug resistance. In fact, miRNA-based anticancer therapies are being developed, either alone or in combination with targeted therapies, with the goal to improve disease response and increase patient survival.^13^

The protein kinase B (AKT/PKB) kinases, including AKT1, AKT2 and AKT3, are essential regulators of various signaling pathways and cellular processes.^14^ Hyper-activation of AKT kinases have been frequently observed in human cancers.^15^ Activation of AKT requires both translocation to the plasma membrane and phosphorylation at Thr308 and Ser473.^16, 17^ Further studies have demonstrated that Thr308 phosphorylation is necessary and sufficient for AKT activation^18^ and that dephosphorylation at Thr308 alone leads to deactivation of AKT.^19, 20^ X-linked inhibitor of apoptosis protein (XIAP) is a member of the inhibitor of apoptosis proteins (IAPs) family and has a significant role in cell survival by modulating death-signaling pathways at a post-mitochondrial level.^21, 22^ Studies have shown that AKT activation can enhance the protein stability of XIAP, therefore elevating XIAP expression.^23, 24, 25^ Consequently, AKT has been shown to promote cell survival through the XIAP-mediated anti-apoptotic pathway.^26^

The serine/threonine protein phosphatase 2 A (PP2A) holoenzyme is composed of a catalytic C subunit, a structural A subunit and a regulatory B subunit. PPP2R1B or PP2A A subunit beta isoform (PP2A-A*β*) is a constant regulatory subunit of PP2A required to activate PP2A. PPP2R1B was initially characterized as a tumor suppressor. It is located at a chromosomal region (11q23) frequently deleted in human cancers.^27^ Its mutations and alterations have been found in colorectal and other cancers.^28, 29^ Cancer-associated mutants of PPP2R1B have been shown to be incompetent to bind the B and/or C subunits *in vitro*, resulting in PP2A inactivation.^30, 31^ PP2A regulates numerous signaling pathways. Specifically, PP2A has an important role in regulating AKT activity by dephosphorylating AKT at Thr308 and Ser473, leading to AKT inactivation.^19, 32, 33, 34^

In this study, we have discovered a novel miR-587/PPP2R1B (PP2A)/pAKT/XIAP signaling axis that mediates the response of colon cancer cells to 5-FU treatment. Our results show that miR-587 expression is suppressed by 5-FU treatment in the sensitive but not resistant colon cancer cells. MiR-587 confers resistance to 5-FU-induced apoptosis through the inhibition of PPP2R1B expression, which is a direct target of miR-587. Knockdown of PPP2R1B by siRNAs confers 5-FU resistance in colon cancer cells, mimicking miR-587 effect. Inhibition of miR-587 expression or rescue of PPP2R1B expression in colon cancer cells increases their sensitivity to 5-FU treatment. Additionally, an AKT inhibitor, MK-2206, re-sensitizes miR-587-expressing cells to 5-FU treatment. Moreover, experiments in tumor xenograft mouse models reveal that miR-587 significantly reduces the effectiveness of 5-FU in the inhibition of tumor growth *in vivo*. Importantly, studies of colorectal cancer specimens indicate a positive correlation between miR-587 expression and chemoresistance and an inverse correlation between PPP2R1B expression and drug resistance. Our studies have identified miR-587 as a potential target for drug resistance in colorectal cancer and suggested that modulating the PPP2R1B (PP2A)/pAKT/XIAP axis may have benefits against drug resistance.

## Results

### MiR-587 mediates resistance to 5-FU-induced apoptosis in colon cancer cells *in vitro*

**Figure 1:**
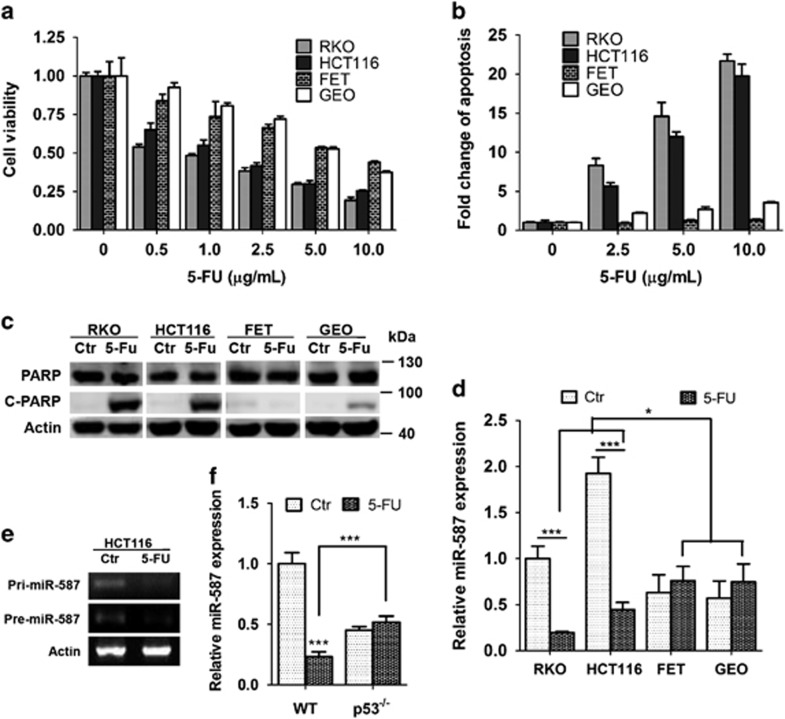
Colon cancer cells show differential responses to 5-FU treatment. (**a** and **b**), RKO, HCT116, FET and GEO cells were treated with increasing concentrations of 5-FU for 72 h. MTT assays (**a**) and DNA fragmentation assays (**b**) showed different sensitivity of those cells to 5-FU treatment. (**c**) Western blot analysis showed that cleaved PARP was higher in RKO and HCT116 cells than in FET and GEO cells after 10 *μ*g/ml of 5-FU treatment for 72 h. (**d**) Determined by real time Q-PCR analysis, miR-587 expression in RKO and HCT116 cells, which are relatively sensitive to 5-FU-induced apoptosis, is significantly reduced after exposure to 8 *μ*g/ml 5-FU for 72 h, whereas its expression remained unchanged in relatively resistant FET and GEO cells. (**e**) RT-PCR analysis showed 5-FU inhibited the expression of pri-miR-587 and pre-miR-587. (**f**) Quantitative real-time PCR analysis showed 5-FU inhibited miR-587 expression in wild-type (WT) p53 but not p53^−/−^ HCT116 cells. The data are presented as the mean±S.D. of triplicate experiments. *P<0.05, ***P<0.001

5-FU is one of the most important chemotherapeutic agents for colorectal cancer treatment. However, intrinsic or acquired drug resistance of cancer cells has been a major obstacle. To understand the mechanisms of drug resistance, we determined 5-FU effect in a panel of colon cancer cell lines, in which 5-FU decreased cell viability in a dose-dependent manner (Figure 1a). 5-FU effect was much more pronounced in RKO and HCT116 cells than in FET and GEO cells. Similarly, RKO and HCT116 cells displayed much higher sensitivity to 5-FU-induced apoptosis than FET and GEO cells as demonstrated by DNA fragmentation assays (Figure 1b) and PARP cleavage (Figure 1c). To identify the molecular determinants of differential 5-FU responses, we evaluated the contribution of miRNAs. Through a functional screening of an miRNA library, we have identified potential miRNAs whose expression conferred 5-FU resistance in colon cancer cells (Zhang *et al.*, unpublished data). Among those identified miRNAs, the expression of miR-587 was significantly suppressed by 5-FU in highly sensitive RKO and HCT116 cells, but not in relatively resistant FET and GEO cells (Figure 1d). Although the basal level of miR-587 expression was higher in RKO and HCT116 cells than in FET and GEO cells in the absence of 5-FU, 5-FU treatment reduced the level in RKO and HCT116 cells to lower than that in both control and treated FET and GEO cells (Figure 1d, *P<0.05). In addition, the expression of both pri-miR-587 and pre-miR-587 were inhibited by 5-FU in HCT116 cells (Figure 1e), suggesting that 5-FU might regulate miR-587 expression at the transcriptional level.

Given that FET cells bear mutated p53,^35^ whereas HCT116 and RKO cells express wild-type p53,^35, 36^ these results suggested that p53 may have a role in 5-FU-mediated suppression of miR-587 expression. Treatment of p53 wild-type and p53^−/−^ HCT116 cells with 5-FU resulted in the inhibition of miR-587 expression in p53 wild-type but not p53^−/−^ HCT116 cells (Figure 1f), indicating that 5-FU-mediated inhibition of miR-587 expression is p53-dependent.

**Figure 2:**
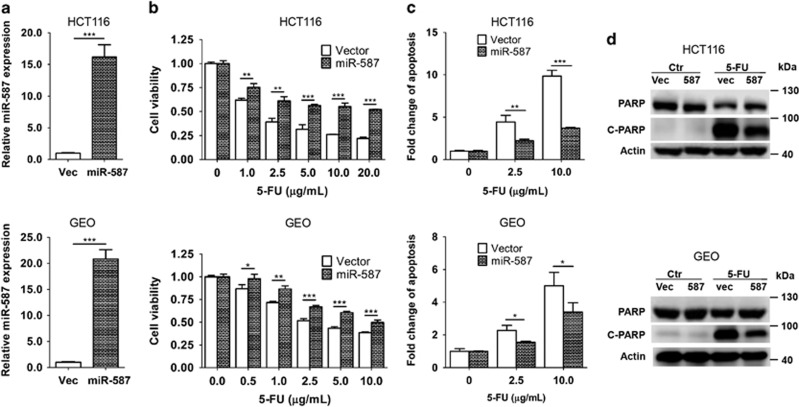
MiR-587 confers resistance to 5-FU treatment in colon cancer cells *in vitro*. (**a**) miR-587 expression was increased 16–20-fold in HCT116 and GEO cells as compared with vector control, and ectopically expressing miR-587 as determined by quantitative real-time PCR. (**b** and **c**) miR-587- and vector-expressing cells were treated with increasing concentrations of 5-FU for 72 h. MTT assays (**b**) and DNA fragmentation assays (**c**) showed increased resistance to 5-FU-induced apoptosis in miR-587-expressing cells compared with the control cells. (**d**) Western blot analysis showed that cleaved PARP was lower in miR-587-expressing cells than vector control cells after 5-FU treatment (HCT116, 10 *μ*g/ml; GEO, 20 *μ*g/ml) for 72 h. The data are presented as the mean±S.D. of triplicate experiments. *P<0.05, **P<0.01, ***P<0.001

To determine whether miR-587 contributes to 5-FU resistance, it was ectopically expressed in HCT116 and GEO cells, resulting in 16- and 20-fold increase of miR-587 expression, respectively (Figure 2a). Consequently, miR-587-expressing cells showed increased cell viability (Figure 2b and Supplementary Figure S1A) and decreased apoptosis (Figure 2c) in response to 5-FU compared with vector control cells with a more pronounced effect in HCT116 cells than in GEO cells. Although GEO cells were relatively resistant to the 5-FU effect, increased expression of miR-587 further enhanced their 5-FU resistance. Consistently, induction of PARP cleavage by 5-FU was reduced in miR-587-expressing cells as compared with vector control cells (Figure 2d). These results indicate that miR-587 protected HCT116 and GEO cells from 5-FU-induced apoptosis.

**Figure 3:**
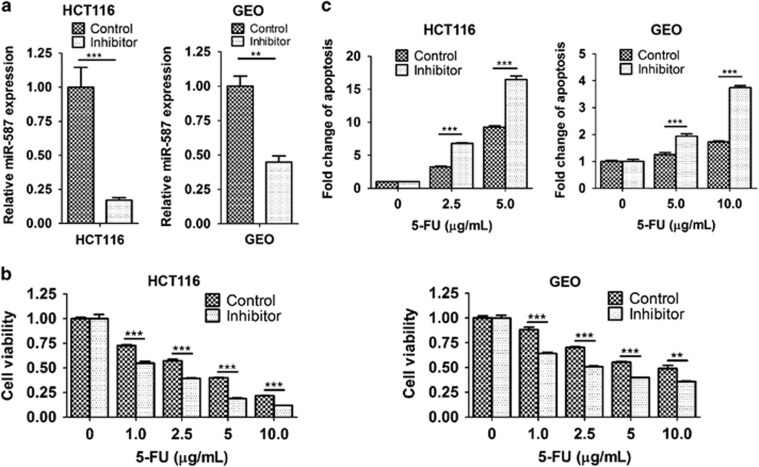
Inhibition of miR-587 sensitized colon cancer cells to 5-FU-induced apoptosis. (**a**) An miR-587 inhibitor decreased miR-587 expression in HCT116 and GEO cells as determined by quantitative real-time PCR. (**b** and **c**) The miR-587 inhibitor-treated or control cells were subjected to increasing concentrations of 5-FU for 72 h. MTT assays (**b**) and DNA fragmentation assays (**c**) showed increased sensitivity to 5-FU-induced apoptosis in the inhibitor-treated cells as compared with the control cells. The data are presented as the mean±S.D. of triplicate experiments. **P<0.01, ***P<0.001

Complementarily, an anti-hsa-miR-587 miScript miRNA inhibitor, chemically synthesized, single-stranded, modified RNA, was used to inhibit miR-587 expression and function. As shown in Figure 3a, the inhibitor decreased miR-587 expression effectively in both HCT116 and GEO cells. As a result, the inhibitor sensitized HCT116 and GEO cells to 5-FU treatment as reflected by reduced cell viability (Figure 3b and Supplementary Figure S1B) and increased apoptosis (Figure 3c and Supplementary Figure S1C). Taken together, these results demonstrate that miR-587 confers resistance to 5-FU-induced apoptosis in colon cancer cells.

### MiR-587 reduces the efficiency of 5-FU-induced inhibition of tumor growth in a colon cancer xenograft model *in vivo*

To determine whether miR-587 conferred resistance to 5-FU-induced apoptosis *in vitro* translates to drug resistance *in vivo*, we used a colon tumor xenograft model described previously^37^ for this study. Briefly, two million HCT116 cells expressing the control vector or miR-587 were subcutaneously inoculated into athymic nude mice. Mice were randomly divided into two groups 7 days after innoculation. One group was treated with 5-FU (40 mg/kg per day) administered by i.p. injection and the other with the carrier. The mice were treated for five consecutive days per week for 2 weeks.^37, 38^ Tumor growth and therapeutic response were monitored during the course of 5-FU treatment.

**Figure 4:**
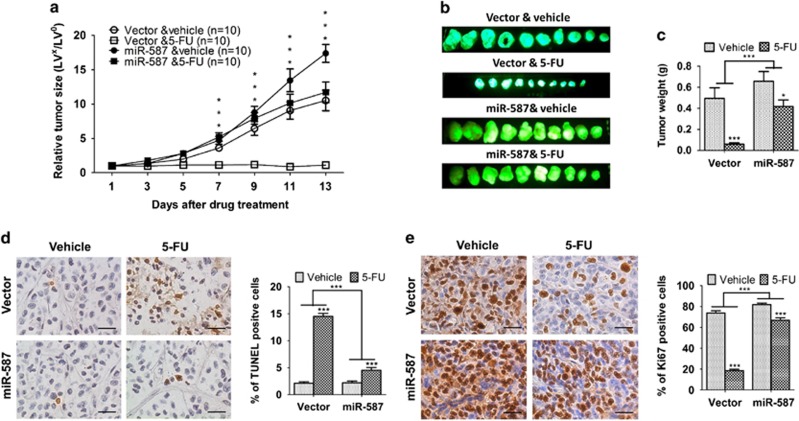
MiR-587 decreases the effectiveness of 5-FU in the inhibition of tumor growth *in vivo*. Xenograft tumor growth curves (**a**), pictures of tumors taken on the same scale (**b**) and tumor weights (**c**) are shown. (**d** and **e**) Images of TUNEL (**d**) and Ki67 (**e**) staining of tumors are shown in the left panels. The images are representative of multiple fields of tumor sections from each group. Percentage of positive TUNEL (**d**) and Ki67 (**e**) staining cells were determined (right panels). The data are presented as the mean±S.E. *P<0.05, ***P<0.001. Scale bars, 25 *μ*m

Xenograft tumor growth curves showed that miR-587 conferred the tumors growth advantage as compared with vector control (Figure 4a). More importantly, miR-587 induced a marked difference in the response of tumors to 5-FU treatment. Tumors with vector-expressing cells (designated as vector tumors) were very sensitive to 5-FU treatment and failed to grow during drug treatment, whereas tumors with miR-587-expressing cells (designated as miR-587 tumors) were relatively resistant to 5-FU treatment and continued to grow at a steady rate (Figure 4a). In addition, compared with vector tumors, miR-587 tumors showed considerably smaller reduction of tumor size (Figure 4b) and tumor weight (Figure 4c) after 5-FU treatment. These results indicated that 5-FU treatment was much less potent in the inhibition of tumor growth of miR-587-expressing cells than that of vector control cells. To determine whether miR-587-mediated resistance to apoptosis *in vitro* was associated with decreased 5-FU effect *in vivo*, TUNEL assays were performed to examine the apoptotic index of tumors. TUNEL staining showed that the percentage of apoptotic cells was similar in miR-587 and vector tumors. However, the increase of apoptotic cells triggered by 5-FU treatment was considerably lower in miR-587 tumors than in vector tumors (2.1- *versus* 6.8-fold, Figure 4d). Moreover, Ki67 staining showed that miR-587 tumors have slightly more proliferative cells than vector tumors in the absence of 5-FU (Figure 4e), which may partially explain the small increase of growth rate of miR-587 tumors (Figure 4a). In addition, 5-FU treatment decreased the percentage of proliferative cells in miR-587 tumors by 20% and in vector tumors by 75% (Figure 4e), indicating that miR-587 tumors were more resistant to 5-FU-mediated inhibition of cell proliferation than vector tumors. These studies suggest that the effect of miR-587 on drug resistance was a combined result of its resistance to 5-FU-induced apoptosis and inhibition of cell proliferation. Taken together, these *in vitro* and *in vivo* results reveal a critical role of miR-587 in drug resistance of colon cancer cells.

### MiR-587 mediates drug resistance by regulating the expression of its target gene PPP2R1B

**Figure 5:**
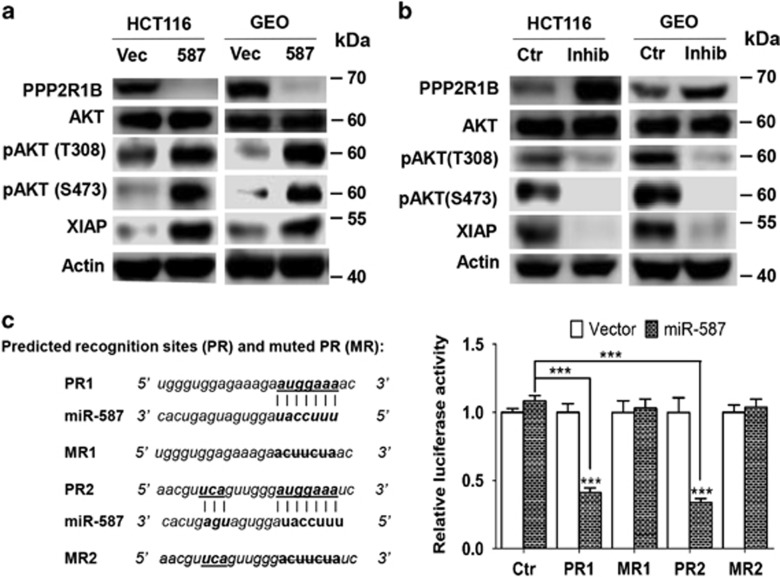
PPP2R1B is a direct target of miR-587. (**a**) PPP2R1B expression was decreased and AKT phosphorylation and XIAP expression were increased by miR-587 expression in HCT116 and GEO cells as determined by western blot analysis. (**b**) The miR-587 inhibitor increased PPP2R1B expression and suppressed AKT phosphorylation and XIAP expression, determined by western blot analysis. (**c**) The miR-587 recognition sites, PR1 and PR2, along with their respective mutants, MR1 and MR2, are shown in the left panel. Luciferase constructs, psiCHECK2-PR1 and psiCHECK2-PR2, their mutants, psiCHECK2-MR1 and psiCHECK2-MR2, and control plasmid psiCHECK2 (Ctr) were transfected into miR-587-expressing or vector control HCT116 cells. Dual luciferase assays were performed and relative luciferase activity was determined (right panels). The data are presented as the mean±S.D. of triplicate experiments. ***P<0.001

To identify target genes of miR-587, we applied several algorithms that predict the mRNA targets of miRNAs – TargetScan,^39^ PicTar^40^ and miRanda-mirSVR.^41^ The candidate target genes were predicted based on the representation of miR-587 recognition sites in their 3′-UTRs. Among those examined, PPP2R1B, a regulatory subunit of PP2A, showed decreased expression in HCT116 and GEO cells expressing miR-587 as compared with vector cells (Figure 5a). On the other hand, the miR-587 inhibitor increased PPP2R1B expression in both cell types (Figure 5b). To determine whether PPP2R1B is a direct target of miR-587, oligos representing two predicted recognition sites of miR-587 in the 3′-UTR of PPP2R1B were synthesized and cloned into the luciferase reporter construct psiCHECK2 (designated psiCHECK2-PR1 and -PR2), whereas oligos with seed sequences of the recognition sites mutated were also cloned into the same construct (designated psiCHECK2-MR1 and -MR2), which were used as controls (Figure 5c, left panel). Luciferase assays showed that the luciferase activity of psiCHECK2-PR1 and -PR2 was decreased in miR-587-expressing cells as compared with vector control cells, whereas that of the control plasmid, psiCHECK2, and of the mutant constructs, psiCHECK2-MR1 and -MR2, remained unchanged (Figure 5c, right panel). These results indicate that miR-587 represses the 3′-UTR of PPP2R1B and that the expression of PPP2R1B is directly regulated by miR-587.

**Figure 6:**
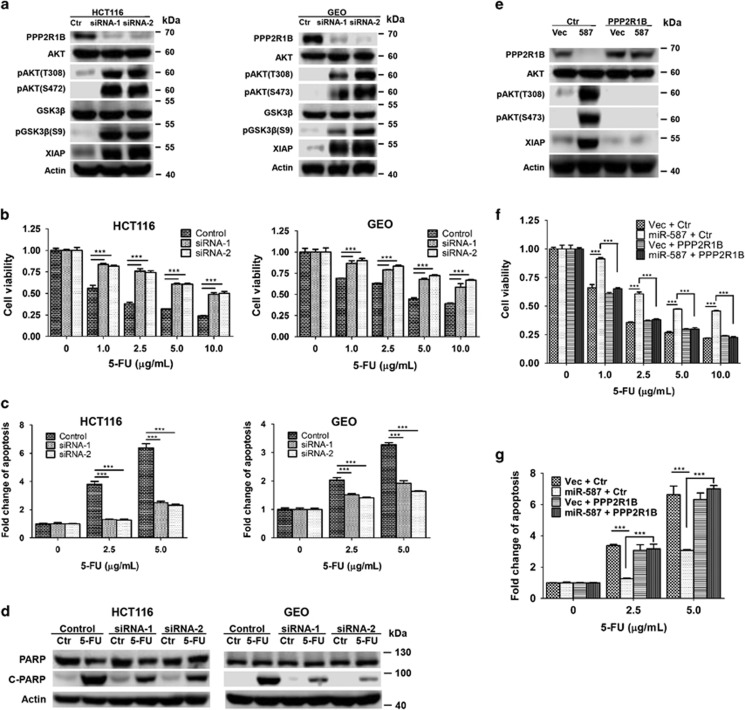
PPP2R1B expression is required for 5-FU-induced apoptosis. (**a**) Expression of PPP2R1B was knocked down by siRNAs in HCT116 and GEO cells, resulting in the upregulation of AKT phosphorylation, GSK3*β* phosphorylation and XIAP expression. (**b**, **c** and **d**) PPP2R1B knockdown and control cells were treated with increasing concentrations of 5-FU for 72 h. MTT assays (**b**), DNA fragmentation assays (**c**) and western blot analysis of cleaved PARP (**d**) (5-FU treatment, HCT116, 10 *μ*g/ml; GEO, 20 *μ*g/ml) were performed, all of which indicated that knockdown of PPP2R1B expression conferred resistance to 5-FU-induced apoptosis. (**e**) PPP2R1B was ectopically expressed in HCT116 vector- and miR-587-expressing cells. Western blot analysis was performed to confirm the rescued expression of PPP2R1B. As a result, AKT phosphorylation and XIAP expression were inhibited. (**f** and **g**) Cells were treated with increasing concentrations of 5-FU for 72 h. MTT assays (**f**) and DNA fragmentation assays (**g**) showed that restoration of PPP2R1B expression re-sensitized miR-587-expressing cells to 5-FU treatment. The data are presented as the mean±S.D. of triplicate experiments. ***P<0.001

PPP2R1B has been characterized as a tumor suppressor.^28^ As a constant regulatory subunit of the PP2A complex, PPP2R1B is crucial to the function of PP2A, a well-established phosphatase regulating numerous signaling pathways involved in cell proliferation, signal transduction and apoptosis.^42^ To determine whether PPP2R1B modulates 5-FU-induced apoptosis, its expression was knocked down by two independent siRNAs, which demonstrated robust effect in HCT116 and GEO cells, resulting in at least 90% reduction of PPP2R1B expression (Figure 6a). Knockdown of PPP2R1B expression enhanced the tolerance of HCT116 and GEO cells to 5-FU-induced apoptosis as demonstrated by increased cell viability (Figure 6b and Supplementary Figure S1D) and reduced apoptosis (Figure 6c and Supplementary Figure S1E and Figure 6d). These results indicated that PPP2R1B was required for 5-FU-induced apoptosis, suggesting that miR-587 may increase 5-FU resistance through the inhibition of PPP2R1B expression.

We next determined whether miR-587 mediated resistance to 5-FU-induced apoptosis could be reversed by restoration of PPP2R1B expression. PPP2R1B cDNA was introduced into miR-587-expressing HCT116 cells. Ectopically expressed PPP2R1B is resistant to downregulation mediated by miR-587 owing to the lack of 3′-UTR (Figure 6e). Restoration of PPP2R1B expression almost completely restored the sensitivity of HCT116 cells to 5-FU-induced apoptosis as reflected by cell viability (Figure 6f and Supplementary Figure S1F) and apoptosis (Figure 6g and Supplementary Figure S1G) assays. These results indicate that miR-587 enhances 5-FU resistance through the downregulation of PPP2R1B expression.

### AKT activation mediated by PPP2R1B contributes to miR-587-conferred 5-FU resistance in colon cancer cells

It has been shown that AKT is an anti-apoptotic factor and that its activation is negatively regulated by the PP2A complex through dephosphorylation of AKT.^19, 32, 33, 34^ One of the anti-apoptotic effectors regulated by AKT is XIAP.^43, 44, 45^ To determine the underlying mechanisms of miR-587-mediated 5-FU resistance, we examined AKT/XIAP signaling and found that miR-587 increased AKT phosphorylation at both Thr308 and Ser473 and upregulated XIAP expression in HCT116 and GEO cells (Figure 5a) and that the miR-587 inhibitor suppressed AKT phosphorylation at both sites and reduced XIAP expression (Figure 5b). In addition, the knockdown of PPP2R1B expression significantly increased AKT phosphorylation and AKT activation, as reflected by the phosphorylation of GSK3*β*, a well-characterized AKT substrate,^46^ and enhanced XIAP expression (Figure 6a). In contrast, re-expression of PPP2R1B inhibited AKT phosphorylation and activation, and XIAP expression (Figure 6e). These results support the possible involvement of AKT/XIAP in miR-587-mediated 5-FU resistance.

**Figure 7:**
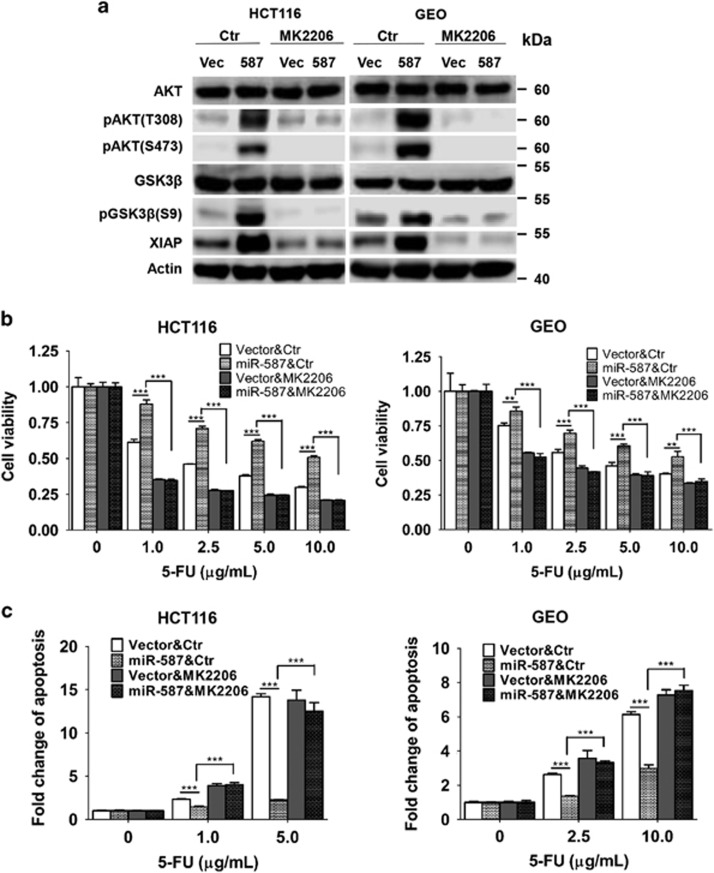
An AKT inhibitor, MK2206, sensitizes miR-587-expressing cells to 5-FU-induced apoptosis. (**a**) AKT phosphorylation, GSK3*β* phosphorylation and XIAP expression were downregulated by MK2206 in miR-587-expressing cells as examined by western blot analysis. (**b** and **c**) HCT116 and GEO cells were treated with MK2206 (HCT116, 0.5 *μ*M; GEO, 1.25 *μ*M) together with 5-FU for 72 h. MTT assays (**b**) and DNA fragmentation assays (**c**) were performed, which showed that MK2206 re-sensitized miR-587-expressing cells to 5-FU treatment. The data are presented as the mean±S.D. of triplicate experiments. **P<0.01, ***P<0.001

To confirm that AKT contributes to miR-587-mediated 5-FU resistance, a specific and potent AKT inhibitor, MK2206,^47, 48^ was used to block AKT activation. Although MK2206 had little effect on AKT phosphorylation in vector control cells, it significantly repressed AKT phosphorylation at both Thr308 and Ser473 and GSK3*β* phosphorylation in miR-587-expressing cells (Figure 7a). In addition, MK2206 also reduced XIAP expression in both cell types, with more obvious reduction in miR-587-expressing cells (Figure 7a). As a result, it diminished miR-587-conferred 5-FU resistance in both HCT116 and GEO cells, as demonstrated by cell viability (Figure 7b and Supplementary Figure S1H) and apoptosis (Figure 7c and Supplementary Figure S1I) assays. Taken together, these results indicate that miR-587 mediates resistance to 5-FU-induced apoptosis through regulating the PPP2R1B (PP2A)/pAKT/XIAP axis.

### Expression of miR-587 and PPP2R1B is positively and inversely correlated with chemoresistance, respectively, in colorectal cancer patients

**Figure 8:**
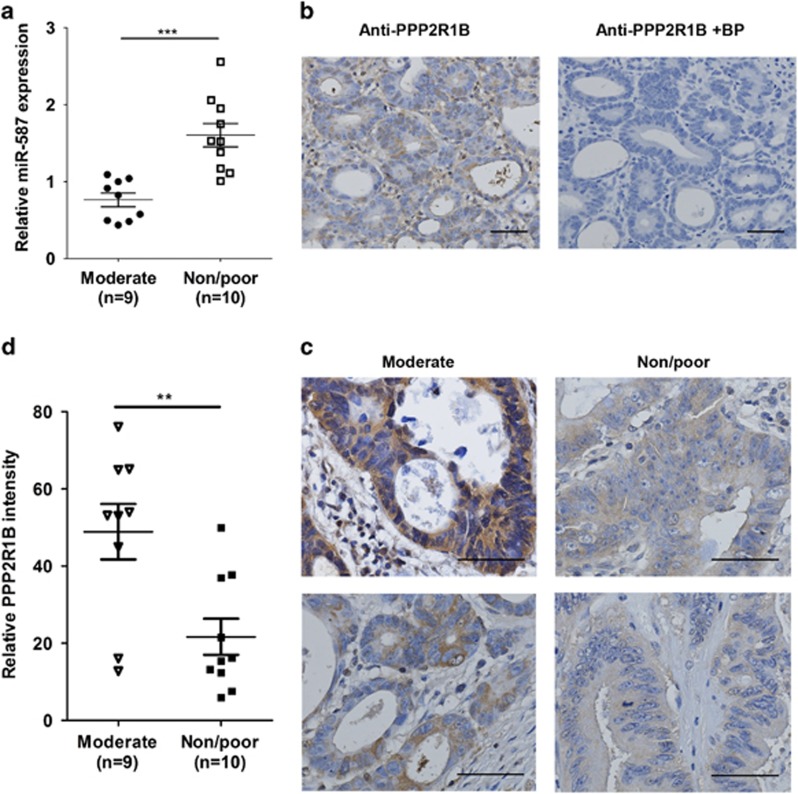
Expression of miR-587 and PPP2R1B positively and inversely correlate with chemoresistance in human colorectal cancer specimens. (**a**) RNA samples prepared from 9 moderately and 10 non-responding or poorly responding colorectal tumors were used to determine miR-587 expression. The data are presented as the mean±S.E. ***P<0.001. (**b**) IHC staining of a colorectal tumor with a PPP2R1B antibody in the absence (left) or presence (right) of a specific blocking peptide (BP) is shown, indicating the specificity of the anti-PPP2R1B antibody. Scale bars, 50 *μ*m. (**c** and **d**) IHC staining of PPP2R1B was performed in sections prepared from colorectal tumors described above. (**c**) Representative images of IHC staining of PPP2R1B in each group are shown. Scale bars, 50 *μ*m. (**d**) Quantification of staining intensity of PPP2R1B was performed. Error bars indicate S.E.M. of the values in each group. **P<0.01

To determine the clinical relevance of miR-587 expression in chemoresistance of colorectal cancer patients, we extended our analyses by quantifying miR-587 expression in human colorectal adenocarcinoma specimens. RNA was isolated from sections prepared from 19 patients who had received neoadjuvant chemoradiotherapy. Among them, 9 patients had moderate response and the other 10 had no or poor response. As shown in Figure 8a, quantitative real-time PCR assays showed that the expression of miR-587 in non-responders or poor responders was more than two fold of that in moderate responders (***P<0.001).

We further analyzed PPP2R1B expression in these samples using immunohistochemistry (IHC) staining. The specificity of an anti-PPP2R1B antibody was first verified using a specific blocking peptide. As shown in Figure 8b, the blocking peptide completely abolished the staining by the antibody, indicating the specificity of the antibody. IHC staining was then performed on paraffin sections prepared from 19 patients described above. Samples from moderate response and non-response or poor response groups showed different PPP2R1B staining intensity (Figure 8c). Quantification of the staining indicated that the intensity of PPP2R1B staining is approximately 2.5-fold higher in moderate responders than in non-responders or poor responders (Figure 8d, **P<0.01). These results indicate that expression of miR-587 and PPP2R1B positively and inversely correlates with chemoresistance, respectively, in colorectal cancer patients. Taken together with *in vitro* and *in vivo* results, our studies demonstrate that miR-587/PPP2R1B has an important role in drug resistance of colorectal cancer.

## Discussion

We have identified a novel miR-587/PPP2R1B(PP2A)/pAKT/XIAP signaling axis that regulates the response of colon cancer cells to 5-FU treatment. Ectopic expression of miR-587 enhances 5-FU resistance *in vitro* and in tumor xenografts *in vivo*. An miR-587 inhibitor sensitizes colon cancer cells to 5-FU treatment. MiR-587 promotes drug resistance by the inhibition of PPP2R1B expression, which leads to increased AKT activation and XIAP expression, and PPP2R1B is required for 5-FU-induced apoptosis. Importantly, miR-587 expression positively and PPP2R1B expression inversely correlate with drug resistance in colorectal cancer patients. Despite advances in chemotherapy, drug resistance is still one of the biggest obstacles for treating advanced colorectal cancer effectively.^49^ Therefore, the discovery of miR-587 as a contributing factor of drug resistance and the identification of the regulatory miR-587/PPP2R1B (PP2A)/pAKT/XIAP signaling axis may facilitate to design strategies to increase the overall efficacy of chemotherapy of colorectal cancer.

AKT is a major survival factor in colon cancer cells.^50, 51^ Activated AKT mediates cell survival through different mechanisms including blockage of the release of cytochrome c from the mitochondria,^52^ and phosphorylation mediated inactivation of the proapoptotic factors BAD, procaspase-9^53^ and FOXO (forkhead box class O) transcription factors.^54^ In addition, activated AKT also stabilizes XIAP.^23, 24, 25, 26^ XIAP has been shown to be a key determinant for chemoresistance in different types of cancer.^55, 56, 57^ On the basis of the results of this study, we report that the inhibition of AKT activation by MK2206 results in reduced expression of XIAP and increased sensitivity to 5-FU-induced apoptosis (Figure 7), implicating AKT as a contributor to 5-FU resistance through upregulation of XIAP expression. PP2A is a well-established phosphatase and a critical negative regulator of AKT activity by dephosphorylating AKT at both Thr308 and Ser473.^19, 32, 33, 34^ PPP2R1B is an essential constant regulatory subunit of PP2A, serving as a scaffolding molecule to coordinate the assembly of the PP2A holoenzyme. Our studies indicate that knockdown of PPP2R1B expression increases phosphorylation of AKT at both Thr308 and Ser473, which leads to increased resistance to 5-FU treatment (Figure 6). Taken together, these results suggest that miR-587 disrupts PP2A assembly and functions through suppression of PPP2R1B expression, thereby leading to increased AKT activation and elevated XIAP expression, which enhances the downstream anti-apoptotic pathway. Therefore, the combination of an AKT inhibitor with 5-FU may potentially increase efficacy of 5-FU treatment in colorectal cancer patients.

PPP2R1B has been identified as a tumor suppressor in colorectal and lung cancer.^28^ Mutation or deletion of the PPP2R1B gene has been shown to occur in 15% of primary colon tumors, generating a truncated protein that is unable to bind to the catalytic subunit to assemble the PP2A holoenzyme.^28^ It has been reported that PPP2R1B expression is regulated by multiple transcriptional factors including Ets-1, SP1/SP3 and RXRα/*β*.^58^ Our current findings that miR-587 suppresses PPP2R1B expression indicate that miRNA-mediated posttranscriptional regulation could be a novel mechanism for suppressing PPP2R1B expression in colorectal cancer. This is important because cancer cells often develop multiple mechanisms to reduce the expression of or inactivate tumor-suppressor genes. Understanding of the mechanisms would help design strategies to restore the expression or activity of tumor suppressors.

Our discovery of miR-587 as a mediator of drug response in colorectal cancer provides a potential therapeutic target. Inhibition of miR-587 expression or activity could increase colorectal cancer chemosensitivity. Interestingly, we observed that the basal level of miR-587 expression is higher in HCT116 and RKO cells than in FET and GEO cells in the absence of 5-FU (Figure 1d). In fact, miR-587-expressing cells displayed a higher growth rate *in vitro* and *in vivo* than vector-expressing cells without 5-FU treatment (Figure 4a, and data not shown), and HCT116 cells with a relatively high basal level of miR-587 expression are more resistant to growth factor deprivation stress-induced apoptosis than FET cells with a relatively low basal level of miR-587 expression (data not shown). These results indicate that, in addition to 5-FU treatment, miR-587 has a pro-proliferative and pro-survival role under other stress conditions. However, 5-FU decreases miR-587 expression in 5-FU-sensitive HCT116 and RKO cells but not in 5-FU-resistant FET and GEO cells (Figures 1d and e), which leads to lower miR-587 expression in HCT116 and RKO cells than in FET and GEO cells after 5-FU treatment (Figure 1d). This contributes to differential 5-FU response. Therefore, the sensitivity to 5-FU-induced apoptosis is dependent upon the ability of 5-FU to suppress miR-587 expression. 5-FU is often used to treat other types of cancer. Future studies will determine whether the clinical efficacy of 5-FU in treating other tumors is dependent upon its ability to suppress miR-587 expression thereby decreasing drug resistance.

## Materials and methods

### Cell lines and reagents

The human colon carcinoma RKO, HCT116, FET and GEO cell lines were cultured in McCoy's 5 A serum-free medium (Sigma, St Louis, MO, USA) supplemented with 10 ng/ml epidermal growth factor, 20 *μ*g/ml insulin and 4 *μ*g/ml transferrin.^59^ HCT-116 p53^−/−^ cell line was cultured in McCoy's 5 A serum-free medium supplemented with 10% of fetal bovine serum (Life Technologies, Grand Island, NY, USA). Cells were maintained at 37°C in a humidified incubator with 5% CO_2_. 5-FU and MK2206 were purchased from Sigma and Selleckchem (Houston, TX, USA), respectively. Anti-hsa-miR-587 miScript miRNA inhibitor was purchased from Qiagen Inc. (Valencia, CA, USA). Antibodies were purchased as indicated: anti-PPP2R1B (IHC), Santa Cruz Biotechnology Inc. (Santa Cruz, CA, USA); anti-PPP2R1B (western blot), Abcam (Cambridge, MA, USA); anti-PARP, anti-cleaved PARP, anti-AKT, anti-pAKT(T308), anti-pAKT(S473), anti-GSK3*β*, anti-pGSK3*β*(S9) anti-XIAP, Cell Signaling Technology (Beverly, MA, USA); and anti-actin, Sigma.

### Cell viability and apoptosis assays

Colon cancer cells were plated in 96-well plates and treated with 5-FU or MK-2206 for the indicated time. Cells were stained for 2 h with thiazolyl blue tetrazolium bromide (MTT) (Sigma). The OD at 570 nm was read on a ELx808 Absorbance Microplate Reader (BioTek, Winooski, VT, USA) after dissolving in DMSO (Sigma). Cell viability was calculated as a ratio of OD values of drug-treated samples to those of controls. Apoptosis was detected using a DNA fragmentation ELISA kit (Roche, Indianapolis, IN, USA).

### Western blot analysis, RT-PCR and real-time Q-PCR

Whole cell lysates were prepared in RIPA buffer (1% Triton X-100, 1% sodium deoxycholate, 0.1% SDS, 150 mM NaCl, 10 mM Tris–HCl (pH 7.5), 5 mM EDTA and a protease inhibitor cocktail (Sigma)). Equivalent amounts of protein were separated by SDS-PAGE and transferred to a PVDF membrane (Millipore, Billerica, MA, USA). Proteins were detected using an enhanced chemiluminescence system (Amersham Biosciences, Piscataway, NJ, USA).

For RT-PCR, 2 *μ*g of RNA was reverse-transcribed with M-MLV reverse transcriptase (Promega, Madison, WI, USA) using a random primer. cDNA (2 ul) product was used to amplify human pri-miR-587, pre-miR-587 and actin. Primer sequences for pri-miR-587 were 5′-CCAGGCAAGAGAGAGTTGCTG-3′ (forward) and 5′-AGTCACAGGTGCAGACACATT-3′ (reverse), for pre-miR-587 were 5′-TATGCACCCTCTTTCCATAGGTG-3′ (forward) and 5′-ATGGGCTTTCCACTGGTGATG-3′ (reverse), and for actin were 5′-TGACGGGGTCACCCACACTGTGCCCAT-3′ (forward) and 5′-CTAGAAGCATTTGCGGTGGACGATGGAGG (reverse).

Expression of miR-587 was determined by miScript primer assays and miScript SYBR Green PCR Kit from Qiagen Inc. RNU6-2 was used as an endogenous reference gene.

### Luciferase assays

Oligos with sequences of PPP2R1B 3′-UTR containing predicted recognition sites of miR-587 were synthesized and cloned into a Promega psiCHECK-2 vector downstream of Renilla reporter gene to generate psiCHECK2-PR1 and psiCHECK2-PR2. The seed sequences of miR-587 recognition sites were mutated to generate psiCHECK2-MR1 and psiCHECK2-MR2 as negative controls. The psiCHECK2 vector contains a second reporter gene, firefly luciferase, and is designed for endpoint lytic assays. The reporters were transfected into cells using Lipofectamine LTX (Life Technologies). Luciferase activity was measured 48 h later using Dual-Luciferase Reporter Assay (Promega). Values were normalized with firefly luciferase activity.

### Plasmid construction and lentiviral infection

cDNA encoding human miR-587 precursor (~300 bp) was cloned into pCDH-CMV lentiviral vector purchased from System Biosciences (SBI, Mountain View, CA, USA). PPP2R1B lentiviral expression vector was purchased from GeneCopoeia, Inc. (Rockville, MD, USA). Packaging cells (293) were co-transfected with pPackH1 packaging plasmid mix (SBI) and the lentiviral vectors using Fugene HD (Promega). Viruses were harvested 48 h later to infect target cells.

### Knockdown by siRNAs

SiRNAs used to knock down PPP2R1B expression were purchased from GE Healthcare Dharmacon Inc. (Lafayette, CO, USA). The target sequences for siRNAs are GGAATTAGACAGTGTGAAA (siRNA-1) and GAACCTACTTAAAGACTGT (siRNA-2). The siRNAs were transfected into the cells using Dharmaconfect-2 (GE Healthcare Dharmacon Inc.) following the manufacturer's instruction.

### *In vivo* xenograft model

Experiments involving animals were approved by the University of Nebraska Medical Center Institutional Animal Care and Use Committee. HCT116 cells (2 × 10^6^) expressing miR-587 or an empty vector were injected into the flank of male athymic nude mice (4–5-weeks old). Drug administration, data collection and analyses were performed as described previously.^37^

### TUNEL and Ki67 staining

Formalin-fixed paraffin-embedded tissue blocks of tumors were stained for TUNEL and Ki67 using the procedure described previously.^60^ Three tumors from each group were analyzed. Ten histologically similar fields were randomly selected from each slide for analysis. Apoptosis and proliferation of tumor cells was determined quantitatively by counting the numbers and calculating the percentage of positively stained cells for TUNEL and Ki67 at × 20 magnification, respectively.

### Determination of miR-587 expression in human tissue samples

Formalin-fixed paraffin-embedded blocks of human colorectal adenocarcinomas were obtained from files of Department of Pathology and Microbiology at University of Nebraska Medical Center. The ages of all patients (including both men and women) were between 55 and 85 years. The cancer patients received neoadjuvant chemoradiotherapy prior to surgical removal of the tumors. The study was performed with the approval of the ethics committee (Institutional Review Board).

RNA was extracted using the miRNeasy FFPE kit (Qiagen Inc.) from The formalin-fixed paraffin-embedded slides as described previously.^37^ Expression of miR-587 was determined by Q-PCR as described above.

### IHC staining of PPP2R1B in human tissue samples

IHC staining was performed to examine PPP2R1B expression in tumor samples following ImmunoCruz goat ABC Staining System protocol (Santa Cruz). Briefly, slides were blocked by goat serum after antigen retrieval, followed by incubation with an anti-PPP2R1B antibody for overnight at 4 °C. Slides were then incubated for 30 min with biotinylated secondary antibody, and developed with DAB after incubation with AB enzyme reagent for 30 min. Finally, the sections were counterstained with hematoxylin. For each slide, 10 randomly chosen fields were captured at × 40 magnification. The staining intensity was quantified with Imagescope Software (Aperio: Leica Biosystems Inc., Buffalo Grove, IL, USA).

### Statistical analysis

Statistical analyses were performed using two-way ANOVA or Student's *t*-test.

## Abbreviations

5-FU: 5-fluorouracil
AKT/PKB: protein kinase B
bp: base pair
DNA: deoxyribonucleic acid or deoxyribonucleate
ELISA: enzyme-linked immunosorbent assay
FBS: fetal bovine serum
IHC: immunohistochemistry
miRNA: micro-RNA
mRNA: messenger RNA
PCR: polymerase chain reaction
PVDF: poly(vinylidene difuloride)
PP2A: serine/threonine protein phosphatase 2A
PPP2R1B/PP2A-A*β*: PP2A A subunit beta isoform
RNA: ribonucleic acid or ribonucleate
SDS: sodium dodecyl sulfate
siRNA: small interfering RNA
TUNEL: terminal deoxynucleotidyltransferase-mediated dUTP nick end labeling
UTR: untranslated region
WT: wild type
XIAP: X-linked inhibitor of apoptosis protein

## References

[bib1] Heidelberger C Chaudhuri N Danneberg P Mooren D Griesbach L Duschinsky R Fluorinated pyrimidines, a new class of tumour-inhibitory compounds Nature 1957 179 663 666 13418758 10.1038/179663a0

[bib2] Longley DB Harkin DP Johnston PG 5-fluorouracil: mechanisms of action and clinical strategies Nat Rev Cancer 2003 3 330 338 12724731 10.1038/nrc1074

[bib3] Longley DB Allen WL Johnston PG Drug resistance, predictive markers and pharmacogenomics in colorectal cancer Biochim Biophys Acta 2006 1766 184 196 16973289 10.1016/j.bbcan.2006.08.001

[bib4] Liu Y-Y Han T-Y Giuliano AE Cabot MC Ceramide glycosylation potentiates cellular multidrug resistance FASEB J 2001 15 719 730 11259390 10.1096/fj.00-0223com

[bib5] Longley D Johnston P Molecular mechanisms of drug resistance J Pathol 2005 205 275 292 15641020 10.1002/path.1706

[bib6] Synold TW Dussault I Forman BM The orphan nuclear receptor SXR coordinately regulates drug metabolism and efflux Nat Med 2001 7 584 590 11329060 10.1038/87912

[bib7] Gottesman MM Mechanisms of cancer drug resistance Annu Rev Med 2002 53 615 627 11818492 10.1146/annurev.med.53.082901.103929

[bib8] Bartel DP MicroRNAs: target recognition and regulatory functions Cell 2009 136 215 233 19167326 10.1016/j.cell.2009.01.002 PMC3794896

[bib9] Lewis BP Burge CB Bartel DP Conserved seed pairing, often flanked by adenosines, indicates that thousands of human genes are microRNA targets Cell 2005 120 15 20 15652477 10.1016/j.cell.2004.12.035

[bib10] Lujambio A Lowe SW The microcosmos of cancer Nature 2012 482 347 355 22337054 10.1038/nature10888 PMC3509753

[bib11] Calin GA Sevignani C Dumitru CD Hyslop T Noch E Yendamuri S Human microRNA genes are frequently located at fragile sites and genomic regions involved in cancers Proc Natl Acad Sci USA 2004 101 2999 3004 14973191 10.1073/pnas.0307323101 PMC365734

[bib12] Zhang B Pan X Cobb GP Anderson TA microRNAs as oncogenes and tumor suppressors Dev Biol 2007 302 1 12 16989803 10.1016/j.ydbio.2006.08.028

[bib13] Garzon R Marcucci G Croce CM Targeting microRNAs in cancer: rationale, strategies and challenges Nat Rev Drug Discov 2010 9 775 789 20885409 10.1038/nrd3179 PMC3904431

[bib14] Vivanco I Sawyers CL The phosphatidylinositol 3-kinase–AKT pathway in human cancer Nat Rev Cancer 2002 2 489 501 12094235 10.1038/nrc839

[bib15] Bellacosa A Kumar CC Di Cristofano A Testa JR Activation of AKT kinases in cancer: implications for therapeutic targeting Adv Cancer Res 2005 94 29 86 16095999 10.1016/S0065-230X(05)94002-5

[bib16] Andjelkovic M Alessi DR Meier R Fernandez A Lamb NJ Frech M Role of translocation in the activation and function of protein kinase B J Biol Chem 1997 272 31515 31524 9395488 10.1074/jbc.272.50.31515

[bib17] Bellacosa A Chan TO Ahmed NN Datta K Malstrom S Stokoe D Akt activation by growth factors is a multiple-step process: the role of the PH domain Oncogene 1998 17 313 325 9690513 10.1038/sj.onc.1201947

[bib18] Stokoe D Stephens LR Copeland T Gaffney PR Reese CB Painter GF Dual role of phosphatidylinositol-3, 4, 5-trisphosphate in the activation of protein kinase B Science 1997 277 567 570 9228007 10.1126/science.277.5325.567

[bib19] Beaulieu J-M Sotnikova TD Marion S Lefkowitz RJ Gainetdinov RR Caron MG An Akt/*β*-arrestin 2/PP2A signaling complex mediates dopaminergic neurotransmission and behavior Cell 2005 122 261 273 16051150 10.1016/j.cell.2005.05.012

[bib20] Perrotti D Neviani P Protein phosphatase 2A: a target for anticancer therapy Lancet Oncol 2013 14 e229 e238 23639323 10.1016/S1470-2045(12)70558-2 PMC3913484

[bib21] Dohi T Okada K Xia F Wilford CE Samuel T Welsh K An IAP-IAP complex inhibits apoptosis J Biol Chem 2004 279 34087 34090 15218035 10.1074/jbc.C400236200

[bib22] Dohi T Xia F Altieri DC Compartmentalized phosphorylation of IAP by protein kinase A regulates cytoprotection Mol Cell 2007 27 17 28 17612487 10.1016/j.molcel.2007.06.004 PMC1986705

[bib23] Dan HC Sun M Kaneko S Feldman RI Nicosia SV Wang H-G Akt phosphorylation and stabilization of X-linked inhibitor of apoptosis protein (XIAP) J Biol Chem 2004 279 5405 5412 14645242 10.1074/jbc.M312044200

[bib24] Hatton O Phillips LK Vaysberg M Hurwich J Krams SM Martinez OM Syk activation of phosphatidylinositol 3-kinase/Akt prevents HtrA2-dependent loss of X-linked inhibitor of apoptosis protein (XIAP) to promote survival of Epstein-Barr virus+ (EBV+) B cell lymphomas J Biol Chem 2011 286 37368 37378 10.1074/jbc.M111.255125 PMC3199484 21908615

[bib25] Opel D Naumann I Schneider M Bertele D Debatin K-M Fulda S Targeting aberrant PI3K/Akt activation by PI103 restores sensitivity to TRAIL-induced apoptosis in neuroblastoma Clin Cancer Res 2011 17 3233 3247 21355080 10.1158/1078-0432.CCR-10-2530

[bib26] Shrader M Pino MS Lashinger L Bar-Eli M Adam L Dinney CP Gefitinib reverses TRAIL resistance in human bladder cancer cell lines via inhibition of AKT-mediated X-linked inhibitor of apoptosis protein expression Cancer Res 2007 67 1430 1435 17308080 10.1158/0008-5472.CAN-06-1224

[bib27] Baysal BE Willett-Brozick JE Taschner P Dauwerse J Devilee P Devlin B A high-resolution integrated map spanning the SDHD gene at 11q23: a 1.1-Mb BAC contig, a partial transcript map and 15 new repeat polymorphisms in a tumour-suppressor region Eur J Hum Genet 2001 9 121 129 10.1038/sj.ejhg.5200585 11313745

[bib28] Wang SS Esplin ED Li JL Huang L Gazdar A Minna J Alterations of the PPP2R1B gene in human lung and colon cancer Science 1998 282 284 287 9765152 10.1126/science.282.5387.284

[bib29] Takagi Y Futamura M Yamaguchi K Aoki S Takahashi T Saji S Alterations of the PPP2R1B gene located at 11q23 in human colorectal cancers Gut 2000 47 268 271 10896920 10.1136/gut.47.2.268 PMC1727986

[bib30] Ruediger R Pham HT Walter G Alterations in protein phosphatase 2A subunit interaction in human carcinomas of the lung and colon with mutations in the A beta subunit gene Oncogene 2001 20 1892 1899 10.1038/sj.onc.1204279 11313937

[bib31] Tamaki M Goi T Hirono Y Katayama K Yamaguchi A PPP2R1B gene alterations inhibit interaction of PP2A-A*β* and PP2A-C proteins in colorectal cancers Oncol Rep 2004 11 655 659 14767517

[bib32] Ivaska J Nissinen L Immonen N Eriksson JE Kähäri V-M Heino J Integrin α2*β*1 promotes activation of protein phosphatase 2A and dephosphorylation of Akt and glycogen synthase kinase 3*β* Mol Cell Biol 2002 22 1352 1359 11839802 10.1128/mcb.22.5.1352-1359.2002 PMC134683

[bib33] Li G Ji X-D Gao H Zhao J-S Xu J-F Sun Z-J EphB3 suppresses non-small-cell lung cancer metastasis via a PP2A/RACK1/Akt signalling complex Nat Commun 2012 3 667 22314363 10.1038/ncomms1675

[bib34] Kuo Y-C Huang K-Y Yang C-H Yang Y-S Lee W-Y Chiang C-W Regulation of phosphorylation of Thr-308 of Akt, cell proliferation, and survival by the B55α regulatory subunit targeting of the protein phosphatase 2A holoenzyme to Akt J Biol Chem 2008 283 1882 1892 18042541 10.1074/jbc.M709585200

[bib35] Gayet J Zhou X-P Duval A Rolland S Hoang J-M Cottu P Extensive characterization of genetic alterations in a series of human colorectal cancer cell lines Oncogene 2001 20 5025 5032 11526487 10.1038/sj.onc.1204611

[bib36] Lim HK Bae W Lee HS Jung J Anticancer activity of marine sponge Hyrtios sp. extract in human colorectal carcinoma RKO cells with different p53 status Biomed Res Int 2014 413575 27 10.1155/2014/413575 PMC4163483 25243139

[bib37] Zhang Y Geng L Talmon G Wang J MicroRNA-520 g confers drug resistance by regulating p21 expression in colorectal cancer J Biol Chem 2015 290 6215 6225 25616665 10.1074/jbc.M114.620252 PMC4358260

[bib38] Valeri N Gasparini P Braconi C Paone A Lovat F Fabbri M MicroRNA-21 induces resistance to 5-fluorouracil by down-regulating human DNA MutS homolog 2 (hMSH2) Proc Natl Acad Sci 2010 107 21098 21103 21078976 10.1073/pnas.1015541107 PMC3000294

[bib39] Grimson A Farh KK-H Johnston WK Garrett-Engele P Lim LP Bartel DP MicroRNA targeting specificity in mammals: determinants beyond seed pairing Mol Cell 2007 27 91 105 17612493 10.1016/j.molcel.2007.06.017 PMC3800283

[bib40] Krek A Grün D Poy MN Wolf R Rosenberg L Epstein EJ Combinatorial microRNA target predictions Nature genetics 2005 37 495 500 15806104 10.1038/ng1536

[bib41] Betel D Koppal A Agius P Sander C Leslie C Comprehensive modeling of microRNA targets predicts functional non-conserved and non-canonical sites Genome Biol 2010 11 R90 20799968 10.1186/gb-2010-11-8-r90 PMC2945792

[bib42] Millward TA Zolnierowicz S Hemmings BA Regulation of protein kinase cascades by protein phosphatase 2 A Trends Biochem Sci 1999 24 186 191 10322434 10.1016/s0968-0004(99)01375-4

[bib43] Downward J PI 3-kinase, Akt and cell survival Semin Cell Dev Biol 2004 15 177 182 15209377 10.1016/j.semcdb.2004.01.002

[bib44] Pommier Y Sordet O Antony S Hayward RL Kohn KW Apoptosis defects and chemotherapy resistance: molecular interaction maps and networks Oncogene 2004 23 2934 2949 15077155 10.1038/sj.onc.1207515

[bib45] Chowdhury S Howell GM Rajput A Teggart CA Brattain LE Weber HR Identification of a novel TGF*β*/PKA signaling transduceome in mediating control of cell survival and metastasis in colon cancer PLoS One 2011 6 e19335 21559296 10.1371/journal.pone.0019335 PMC3086924

[bib46] Cross DA Alessi DR Cohen P Andjelkovich M Hemmings BA Inhibition of glycogen synthase kinase-3 by insulin mediated by protein kinase B Nature 1995 378 785 789 8524413 10.1038/378785a0

[bib47] Yang J Wu Z Renier N Simon DJ Uryu K Park DS Pathological axonal death through a MAPK cascade that triggers a local energy deficit Cell 2015 160 161 176 25594179 10.1016/j.cell.2014.11.053 PMC4306654

[bib48] Piovan E Yu J Tosello V Herranz D Ambesi-Impiombato A Da Silva AC Direct reversal of glucocorticoid resistance by AKT inhibition in acute lymphoblastic leukemia Cancer Cell 2013 24 766 776 24291004 10.1016/j.ccr.2013.10.022 PMC3878658

[bib49] Rougier P Van Cutsem E Bajetta E Niederle N Possinger K Labianca R Randomised trial of irinotecan versus fluorouracil by continuous infusion after fluorouracil failure in patients with metastatic colorectal cancer Lancet 1998 352 1407 1412 9807986 10.1016/S0140-6736(98)03085-2

[bib50] Wang J Yang L Yang J Kuropatwinski K Wang W Liu X-Q Transforming growth factor *β* induces apoptosis through repressing the phosphoinositide 3-kinase/AKT/survivin pathway in colon cancer cells Cancer Res 2008 68 3152 3160 18451140 10.1158/0008-5472.CAN-07-5348

[bib51] Wang J Kuropatwinski K Hauser J Rossi MR Zhou Y Conway A Colon carcinoma cells harboring PIK3CA mutations display resistance to growth factor deprivation induced apoptosis Mol Cancer Ther 2007 6 1143 1150 17363507 10.1158/1535-7163.MCT-06-0555

[bib52] Whang YE Yuan X-J Liu Y Majumder S Lewis TD Regulation of sensitivity to TRAIL by the PTEN tumor suppressor Vitam Horm 2004 67 409 426 15110188 10.1016/S0083-6729(04)67021-X

[bib53] Kang KH Kim WH Choi KH p21 promotes ceramide-induced apoptosis and antagonizes the antideath effect of Bcl-2 in human hepatocarcinoma cells Exp Cell Res 1999 253 403 412 10585263 10.1006/excr.1999.4644

[bib54] Greer EL Brunet A FOXO transcription factors at the interface between longevity and tumor suppression Oncogene 2005 24 7410 7425 16288288 10.1038/sj.onc.1209086

[bib55] Miyamoto M Takano M Iwaya K Shinomiya N Kato M Aoyama T X-chromosome-linked inhibitor of apoptosis as a key factor for chemoresistance in clear cell carcinoma of the ovary Br J Cancer 2014 110 2881 2886 24853184 10.1038/bjc.2014.255 PMC4056063

[bib56] Farrand L Byun S Kim JY Im-Aram A Lee J Lim S Piceatannol enhances cisplatin sensitivity in ovarian cancer via modulation of p53, X-linked inhibitor of apoptosis protein (XIAP), and mitochondrial fission J Biol Chem 2013 288 23740 23750 23833193 10.1074/jbc.M113.487686 PMC3745321

[bib57] Gagnon V Van Themsche C Turner S Leblanc V Asselin E Akt and XIAP regulate the sensitivity of human uterine cancer cells to cisplatin, doxorubicin and taxol Apoptosis 2008 13 259 271 18071906 10.1007/s10495-007-0165-6

[bib58] Liu J Ji W Sun S Zhang L Chen HG Mao Y The PP2A-A*β* gene is regulated by multiple transcriptional factors including Ets-1, SP1/SP3, and RXRα/*β* Curr Mol Med 2012 12 982 994 22827437 10.2174/156652412802480916

[bib59] Boyd D Levine A Brattain D McKnight M Brattain M Comparison of growth requirements of two human intratumoral colon carcinoma cell lines in monolayer and soft agarose Cancer Res 1988 48 2469 2474 3281751

[bib60] Geng L Chaudhuri A Talmon G Wisecarver J Are C Brattain M MicroRNA-192 suppresses liver metastasis of colon cancer Oncogene 2013 33 5332 5340 24213572 10.1038/onc.2013.478 PMC4016997

