# Supplementary material for: MicroRNA-587 antagonizes 5-FU-induced apoptosis and confers drug resistance by regulating PPP2R1B expression in colorectal cancer
Source: Cell Death Dis. 2015 Aug 6;6(8):e1845–. doi: 10.1038/cddis.2015.200 (PMC4558495; doi:10.1038/cddis.2015.200)
Supplement: Supplementary Figure S1 [file cddis2015200x1.doc]

**
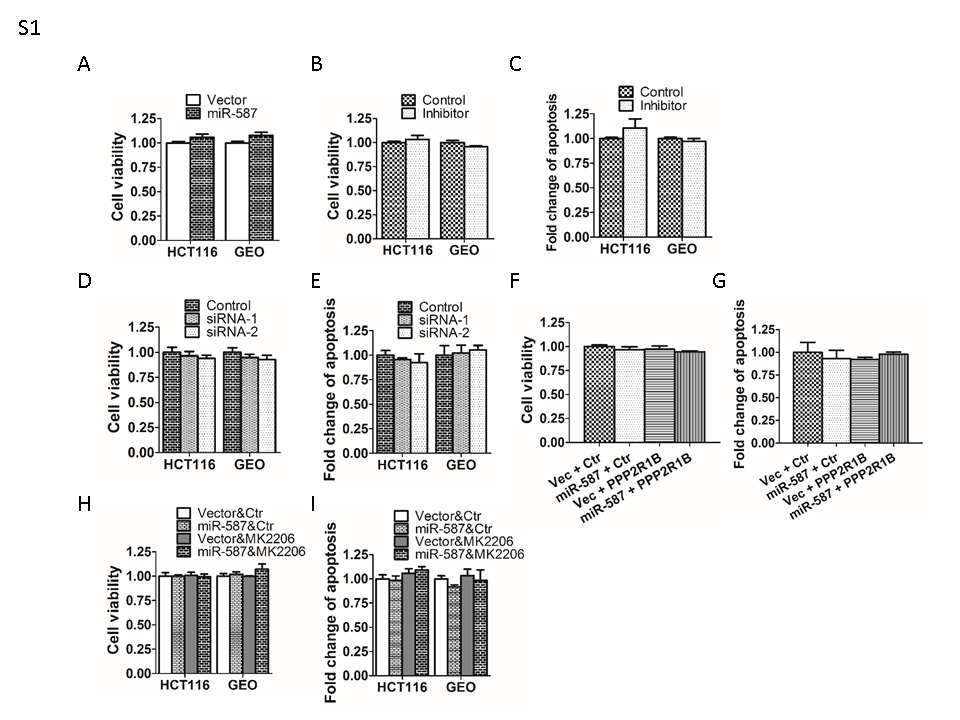
Figure S1.** **Cell viability and apoptosis were compared in the absence of 5-FU treatment.** A, miR-587 was ectopically expressed in HCT116 and GEO cells. Cell viability was compared between vector- and miR-587-expressing cells by MTT assays 72 hrs after plating the cells. B & C, A miR-587 inhibitor was transfected into HCT116 and GEO cells. After 72 hrs, cell viability (B) and apoptosis (C) were compared between control and inhibitor-treated cells by MTT and DNA Fragmentation assays respectively. D & E, Two siRNAs against PPP2R1B were transfected into HCT116 and GEO cells. After 72 hrs, cell viability (D) and apoptosis (E) were compared between control siRNA- and PPP2R1B siRNA-transfected cells by MTT and DNA Fragmentation assays respectively. F & G, PPP2R1B was ectopically expressed in HCT116 vector- and miR-587-expressing cells. After 72 hrs, cell viability (F) and apoptosis (G) were compared using MTT and DNA Fragmentation assays respectively. H & I, HCT116 and GEO cells expressing vector or miR-587 were treated with MK2206 (HCT116, 0.5 µM; GEO, 1.25 µM). After 72 hrs, cell viability (H) and apoptosis (I) were compared using MTT and DNA Fragmentation assays respectively.

.
